# Computational discovery and experimental validation of high–refractive index HfS_2_ nanoresonators

**DOI:** 10.1126/sciadv.adw9339

**Published:** 2025-11-05

**Authors:** Xavier Zambrana-Puyalto, Mark Kamper Svendsen, Amalie H. Søndersted, Avishek Sarbajna, Joakim P. Sandberg, Albert L. Riber, Georgy Ermolaev, Tara Maria Boland, Gleb Tselikov, Valentyn S. Volkov, Kristian S. Thygesen, Søren Raza

**Affiliations:** ^1^ Department of Physics, Technical University of Denmark, Fysikvej, DK-2800 Kongens Lyngby, Denmark.; ^2^ NNF Quantum Computing Programme, Niels Bohr Institute, University of Copenhagen, Denmark.; ^3^ Emerging Technologies Research Center, XPANCEO, Internet City, Emmay Tower, Dubai, United Arab Emirates.

## Abstract

High–refractive index dielectric materials can enhance many optical technologies by enabling efficient light manipulation in waveguides, metasurfaces, and nanoscale resonators. Van der Waals materials, which are anisotropic semiconductor materials, are particularly promising due to their excitonic response and strong in-plane polarizability. Here, we perform ab initio calculations to determine the refractive index of over a hundred anisotropic semiconductor materials, many of them van der Waals in nature. Our computational screening reveals both established and less-explored promising materials, including hafnium disulfide (HfS_2_), which exhibits an in-plane refractive index above 3 and large anisotropy in the visible range. We confirm these properties through ellipsometry and develop a nanofabrication process for HfS_2_, demonstrating Mie-resonant nanodisks. This is achieved by mitigating the air sensitivity of HfS_2_ through storage in controlled environment or encapsulation. Our work provides a comparative overview of high-index van der Waals materials and establishes HfS_2_ as a promising material for visible-range photonics.

## Teaser

Computational discovery of high-index materials leads to HfS_2_ nanodisks that control light at the nanoscale.

## INTRODUCTION

The complex refractive index is a fundamental material property in dielectric nanophotonics that directly influences the performance of optical components such as metasurfaces and optical antennas. A high refractive index with minimal optical absorption is crucial for optimizing these technologies. For instance, metalenses achieve better focusing efficiency and achromatic metasurfaces broaden their operational bandwidth with increasing refractive index (*1*, *2*). Similarly, the quality factor of Mie resonances and supercavity modes in optical resonators scales strongly with the refractive index (*3*, *4*). Moreover, Miller’s rule links a higher refractive index to a stronger second-order nonlinear response, highlighting its importance in nonlinear optics (*5*).

Despite the critical role of the refractive index, current research predominantly relies on materials like silicon and III-V semiconductors (*6*, *7*). These materials offer low-loss operation but are limited to the infrared and red parts of the visible spectrum, constrained by their bandgap energies. Expanding the operational range to the entire visible spectrum requires materials with a larger bandgap energy to avoid optical losses, but this typically results in a lower refractive index. This effect is captured by the so-called Moss rule (*8*, *9*), which shows that there is an inverse relation between the refractive index and the bandgap energy. Consequently, there is a shortage of high–refractive index materials that can operate effectively across the visible spectrum. Identifying and using emerging transparent dielectric materials with high refractive indices across the visible spectrum could substantially enhance the performance and functionality of nanoscale optical devices (*10*).

Bulk van der Waals (vdW) materials are promising in this regard, as they display weak interlayer bonding and strong covalent in-plane bonding as well as host prominent in-plane excitonic transitions (*11*). These key properties result in an increased in-plane refractive index and suggest that vdW materials with large bandgap energies may still have a high refractive index (*10*). Recent measurements have shown that vdW materials, such as transition metal dichalcogenides (TMDCs), have very high in-plane refractive indices in the red and in the near-infrared spectral ranges, surpassing their isotropic counterparts, such as silicon (*12*, *13*). With only few experimentally characterized (*13*, *14*) and more than 1000 theoretically predicted (*15*, *16*), vdW materials could provide the needed material platform to engineer light across the entire visible spectrum.

The search for novel optical materials can be dramatically accelerated using first-principles electronic structure calculations based on density functional theory (DFT). With such methods, it is possible to scrutinize the properties of crystalline materials much more quickly than by traditional experiments, as successfully demonstrated in the areas of catalysis (*17*), batteries (*18*), and solar cells (*19*, *20*). However, apart from a few studies (*21*, *22*), the high-throughput strategy has not been used to search for improved optical materials. The reason for this is that optical properties, such as the refractive index tensor, involve excited electronic states that are orders of magnitude more demanding in terms of computational resources. The current gold standard for first-principles calculation of optical properties is the GW approximation combined with the Bethe-Salpeter Equation (GW-BSE) method (*23*), which accounts for excitonic effects. GW-BSE accurately predicts absorption spectra, but it is very computationally demanding. The high computational cost limits the number of bands that can be included in practice, often leading to an underestimation of the refractive index. To address this, some of us have recently developed the BSE+ method (*24*), which goes beyond the BSE method and substantially improves the agreement with experiments for the refractive index. Specifically, BSE+ improves the convergence of BSE calculations by including transitions outside the active BSE electron-hole subspace at the random phase approximation (RPA) level. Previous efforts to go beyond RPA include density-polarization functional theory (*25*, *26*), whose accuracy depends strongly on the choice of exchange-correlation kernel, often requiring fitting to experimental data. In contrast, BSE+ is an ab initio many-body approach that explicitly accounts for electron-hole interactions. As a result, BSE+ yields refractive index tensors that are in closer agreement with experiment, without relying on kernel fitting.

**Fig. 1.**
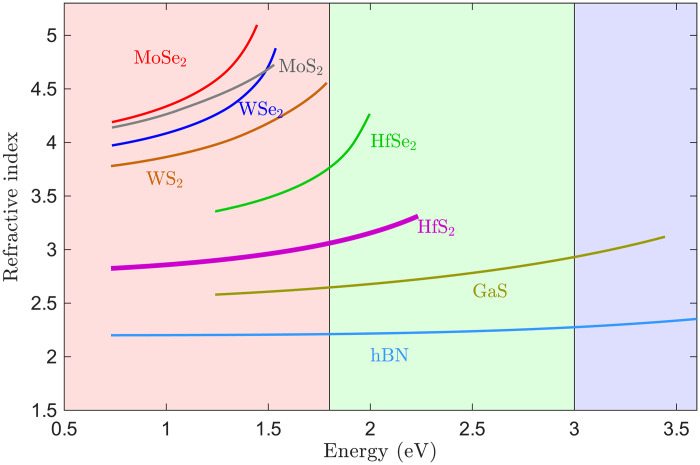
VdW materials in optics. In-plane refractive index for the most commonly used vdW materials in optics along with HfS_2_, which has been identified in this work. Refractive index data are obtained from (*13*, *14*, *37*, *50*, *51*) and plotted in the energy range where the imaginary part of the in-plane refractive index, i.e., the extinction coefficient, is below 0.1. The refractive index of HfS_2_ is obtained by imaging ellipsometry in this work.

In this work, we use high-throughput DFT calculations to screen the optical properties of 338 semiconductor materials, starting from an initial set of 1693 unary and binary materials. Of these semiconductors, 131 have anisotropic refractive indices, and we focus on them because many are intrinsically vdW materials. Our screening identifies many super-Mossian materials (*27*) that surpass the Moss rule, suggesting enhanced refractive index compared to state-of-the-art materials. In particular, the TMDC hafnium disulfide (HfS_2_) stands out due to its low optical losses and its greater than 3 refractive index in the visible spectral range, when compared to other TMDCs used in photonics (Fig. 1). To the best of our knowledge, the optical properties of this material for photonics have not been previously studied. We therefore conduct a thorough experimental optical study of HfS_2_ focusing on both its bulk properties and its behavior when patterned as nanoscale Mie resonators. Using imaging ellipsometry, we measure both the in-plane and out-of-plane complex refractive indices of HfS_2_, confirming the BSE+ calculations of low losses and high refractive index. We also exfoliate HfS_2_ and develop a fabrication procedure to realize HfS_2_ nanodisks that support optical Mie resonances. We observe that HfS_2_ is chemically unstable under ambient conditions, but we show that this instability can be circumvented by storing the material in oxygen-free or humidity-reduced environments, as well as by encapsulating it in hexagonal boron nitride (hBN) or polymethyl methacrylate (PMMA). Overall, our results demonstrate that HfS_2_ has a strong potential for photonic applications due to its high refractive index and low absorption. In addition, our DFT screening provides a comparative overview of promising high-index materials that could be taken further for experimentation.

## RESULTS

### High-throughput screening and refractive index measurement

Our high-throughput screening is based on the procedure developed in our previous work (*22*). Briefly, we extract 1693 elementary and binary materials from the Open Quantum Materials Database (*28*) and relax the atomic structure using DFT with the Perdew-Burke-Ernzerhof (PBE) exchange correlation functional (*29*) and the D3 correction to account for the vdW forces (*30*). Electronic bandgaps for all of the materials are calculated and materials identified as metals are discarded. This leaves 338 semiconductors for which we calculate the refractive index tensor within the RPA. The refractive index tensor of all of these materials are available in the newly established CRYSP database (*31*). Using these data, we compute the fractional anisotropy to categorize the materials based on the anisotropy of their static refractive index tensor. We divide the 338 materials into anisotropic and isotropic. In this study, we focus on the 131 anisotropic materials, as many are vdW in nature and are expected to exhibit higher in-plane refractive indices (*10*) as well as being exfoliable, making them suitable for experimentation (*14*). The remaining 207 isotropic materials are excluded, having already been analyzed in detail in (*22*).

**Fig. 2.**
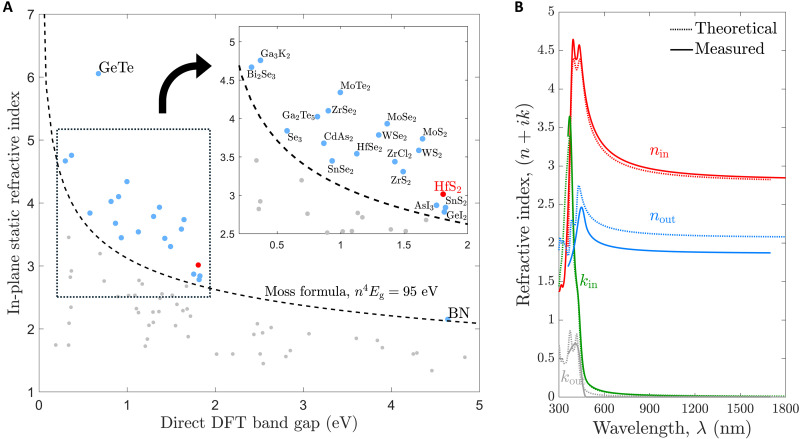
Identification of high–refractive index vdW materials. (**A**) Static in-plane refractive index as a function of direct bandgap energy for 72 anisotropic semiconductor materials along with the Moss formula. Chemical formulas are provided for all of the super-Mossian materials. The inset is a zoomed-in view of the dotted area. (**B**) Computed (dashed lines) and experimentally measured (solid lines) in-plane (red) and out-of-plane (blue) refractive indices of HfS_2_. The in-plane and out-of-plane extinction coefficients are also plotted in green and gray, respectively.

The static in-plane refractive index as a function of the direct bandgap energy of 72 of all 131 anisotropic materials are presented in Fig. 2A. The static refractive index is computed at zero frequency and provides a reasonable estimate of the refractive index in the transparency region of the materials, i.e., at photon energies below the bandgap but above phonon energies. Given our focus on vdW materials, Fig. 2A presents only the 72 uniaxial materials whose two different in-plane refractive index components differ by less than 1%. The remaining 59 materials, which display anisotropy in all three directions, are presented in the Supplementary Text, where we present their three refractive index values $n_{\text{xx}},n_{\text{yy}},n_{\text{zz}}$ as well as their bandgap energy. Figure 2A shows that the refractive index data qualitatively follow the Moss relation, given by $n^{4}E_{g}=95$ eV (*8*). However, there are many materials that surpass the Moss rule, the so-called super-Mossian materials (*27*), which we label by their chemical formulas in Fig. 2A. When examining this map of materials, the PBE functional used in this work is known to systematically underestimate the bandgap energy for certain materials by up to 1 eV or more (*32*). This can be remedied using more accurate and computationally expensive many-body calculation methods, which we present later. Nonetheless, this screening verifies that known high-index vdW materials, such as WS_2_ and MoS_2_, are indeed super-Mossian, and also showcases a wide range of promising but understudied optical materials, such as SnS_2_, ZrS_2_, and HfS_2_. In addition, for photonic applications at telecom energies (0.8 to 0.95 eV), there are many candidate materials that notably surpass traditional semiconductor materials and could enable enhanced performance and miniaturization in, e.g., integrated photonic circuits. In this work, we direct attention to the visible spectrum, where the lack of super-Mossian materials is particularly acute. We therefore focus on the super-Mossian material with the simultaneously highest refractive index and widest bandgap energy, which, based on this screening, is HfS_2_.

To assess the potential of HfS_2_ with high fidelity, we perform a many-body BSE+ calculation of its refractive index tensor [see Methods]. The BSE+ in-plane and out-of-plane complex refractive indices of HfS_2_ are shown in Fig. 2B (dashed lines). We observe that the extinction coefficient *k* of both in-plane and out-of-plane components have values below 0.1 for wavelengths greater than 550 nm. We also see that the theoretical in-plane refractive index $n_{\text{in}}$ is above 3 across the whole visible range, even reaching values of 4.5 in the blue spectral range. The out-of-plane refractive index $n_{\text{out}}$ varies between 2.1 and 2.7 in the visible range, highlighting a strong anisotropy of $n_{\text{in}}-n_{\text{out}}≳1$ . In the Supplementary Text, we show the anisotropy $n_{\text{in}}-n_{\text{out}}$ of the 72 materials shown in Fig. 2A as a function of their bandgap. These theoretical results show that the combination of high-throughput screening combined with many-body BSE+ calculations provide a rational approach to discover new optical materials with high fidelity.

We now turn to an experimental demonstration of HfS_2_, where we first measure the complex refractive index tensor to verify the theoretical predictions. High-purity (>99.995%) HfS_2_ crystals are purchased from a commercial vendor and mechanically exfoliated to produce flakes that are transferred onto a silicon substrate. The crystallinity of the exfoliated flakes is verified with Raman spectroscopy measurements (see Supplementary Text). We measure the refractive index using an imaging ellipsometer [see Methods], and the experimental results (solid lines) are shown along with the theoretical results in Fig. 2B. We observe that the agreement is very good in all the measurements except for the out-of-plane refractive index, where we observe discrepancies in the order of 10%. Nonetheless, we experimentally verify our theoretical predictions, namely that HfS_2_ is a high–refractive index material with very low losses in the visible range.

### Chemical instability

**Fig. 3.**
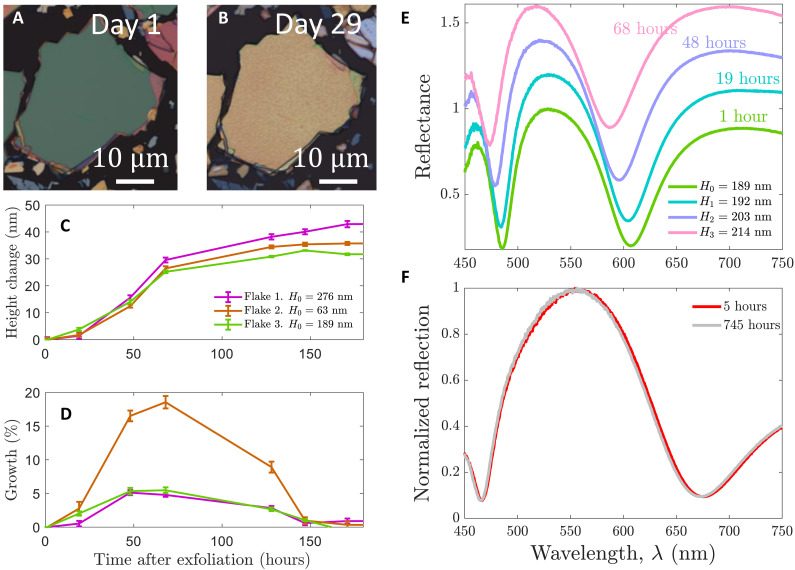
Mitigating the chemical instability of HfS_2_. (**A**) Optical image of a HfS_2_ flake right after exfoliation. (**B**) Optical image of the same HfS_2_ flake after being exposed to ambient conditions for 29 days. Color and structural changes (blisters) are observed. (**C**) Height change (in nanometers) as a function of time (in hours) for three different HfS_2_ flakes. Their initial heights are 275.8, 63.1, and 188.6 nm, respectively. (**D**) Growth (in %) as a function of time after exfoliation (in hours). The growth is computed as increment of height over the current height. (**E**) Reflectance as a function of the wavelength and time after exfoliation for the flake with an initial height *H*_0_ = 188.6 nm. Each curve is displaced by 0.2 units. (**F**) Comparative normalized reflection of a HfS_2_ flake 5 and 745 hours after it was exfoliated. The flake is kept in a desiccator with a humidity reduced to 10%. The reflection data are first divided by the substrate reflection and then normalized to its maximum value. *N* = 1 for all data.

We have observed that the HfS_2_ flakes are chemically unstable under ambient conditions under regular laboratory conditions (Fig. 3, A and B), consistent with previous findings (*33*–*35*). Prior studies have also demonstrated that HfS_2_ can be intentionally converted into hafnia (HfO_2_), thus inducing its thickness to change (*35*, *36*). Because we aim to use HfS_2_ to fabricate nanoscale resonators for optical applications at standard room temperature and under humidity conditions, we investigated the chemical stability of bulk HfS_2_ flakes. For this study, we prepare exfoliated HfS_2_ flakes and measure the initial thickness of three different flakes using atomic force microscopy (AFM). After the initial characterization, we leave the chip under ambient conditions and perform seven new thickness measurements for each flake over the course of 171 hours. The absolute accumulated growth and the relative daily growth of the flakes are shown in Fig. 3 (C and D) respectively. We observe that the three flakes exhibit similar behavior, with rapid growth during the first 3 days, followed by a plateau in daily growth approaching 0% after 6 days. Because of the similar absolute growth, the thinnest flake has the highest daily relative growth, with values of almost 20% in the third day. The thinnest flake also shows the highest accumulated growth of ~56%, while the growth of the two thicker flakes stays in the range of 15 to 17%. These values are well below the observations in (*33*), where an accumulated growth of over 250% was observed for a few-layered HfS_2_ sample.

In addition to thickness changes, we also tracked changes in reflectance of one of the flakes during the first 3 days (Fig. 3E). The reflectance spectra [see Methods for experimental details of the measurement] show pronounced dips due to the excitation of Fabry-Pérot resonances. We observe that the increase in flake thickness is associated with a blueshift of ~20 nm of the Fabry-Pérot resonances. This might seem a bit counterintuitive, as the Fabry-Pérot resonance wavelength $\lambda_{q}$ follows the relation

$$\lambda_{q}=\frac{2\mathrm{nH}}{q}$$ (1)

where *q* is the mode order, *n* is the refractive index, and *H* is the distance between the reflective surfaces of the cavity. In our system, this distance corresponds to the flake thickness. By examining Eq. 1, we observe that Fabry-Pérot resonances should redshift when *H* increases. However, another possibility exists: As the flakes undergo a chemical transformation, their refractive index may be affected. Notably, HfO_2_ has a lower refractive index than HfS_2_ (*37*). We therefore propose that the observed blueshift is a result of the chemically induced decrease in refractive index, which dominates over the increase in thickness.

We observed changes not only in the thickness and in the reflectivity of the HfS_2_ flakes, but also in the structure of the material (Fig. 3, A and B). It is seen that the color of the flake changes, and blisters appear on the surface. Similar blister formation has been reported in previous works (*33*, *36*) and is believed to result from defect points around which oxidation occurs. The exact mechanism underlying the chemical instability of HfS_2_ remains debated. Proposed explanations include a self-limited oxidation process, in which a HfO_2_ layer forms through the reaction of oxygen with the HfS_2_ surface, eventually limiting further oxygen diffusion (*36*). Alternatively, water intercalation has also been suggested to play a role (*35*). In fact, in (*38*), the authors show how an increase of relative humidity from 35 to 70% can have a strong impact in HfS_2_ material degradation. Investigating these mechanisms in detail is beyond the scope of this work. Instead, we adopt a pragmatic approach and try to mitigate the chemical instability of HfS_2_ by exploring different environmental conditions.

To mitigate the chemical instability of HfS_2_ flakes, we first investigated long-term storage of HfS_2_ flakes in both inert argon atmosphere in a glove box as well as a simpler humidity-controlled desiccator. We find that both storage conditions effectively stabilize the material. In particular, we track the reflection [see the Supplementary Materials for details] for a period of 1 month for two samples, where one is stored under ambient conditions and the other one is stored in a desiccator, which reduces the humidity down to 10%. A subset of these results shows that the reflection remains virtually unchanged (Fig. 3F), with detailed findings provided in the Supplementary Text. Another mitigation strategy we have explored is encapsulation. In the Supplementary Materials, we also present a comparative study where we track the reflection of three different samples over 647 hours: (i) HfS_2_ flakes exposed to ambient conditions, (ii) HfS_2_ flakes encapsulated by hBN, and (iii) HfS_2_ flakes spin-coated with PMMA. Our results show that both encapsulation methods substantially reduce the blueshift observed in unprotected HfS_2_ flakes. These findings demonstrate multiple strategies for mitigating chemically induced changes and retaining the desired optical properties of HfS_2_, either through encapsulation or storing the material in a controlled atmosphere.

### Fabrication of optical HfS_2_ nanodisks

**Fig. 4.**
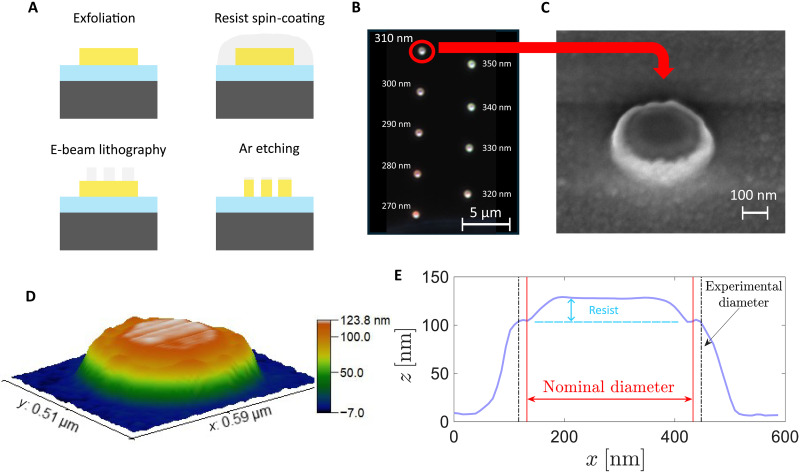
Fabrication of optical HfS_2_ resonators. (**A**) The fabrication process consists of exfoliation, resist spin-coating, electron-beam (e-beam) lithography and argon etching. (**B**) Dark-field optical image at a 100× magnification of a series of HfS_2_ nanoresonators with nominal diameters ranging from 270 to 350 nm in steps of 10 nm. A nanoresonator with a nominal diameter of 310 nm is encircled in red and imaged with scanning electron microscopy (SEM) in (**C**). (**D**) Three-dimensional plot of a height measurement using an AFM. Both (C) and (D) reveal residual resist on top of the nanoresonator and redeposition of HfS_2_ on the sidewalls. (**E**) AFM height measurement along a transverse line for the same resonator as (D), indicating the nominal diameter of 310 nm. The residual resist thickness and actual diameter are also indicated.

Our goal is to leverage the high refractive index and low losses of HfS_2_ to create nanostructures that support optical resonances in the visible range. This work represents a demonstration of lithographic patterning of bulk HfS_2_ flakes. We fabricate 100-nm-thick nanodisks with diameters from 100 to 350 nm, following the procedure schematically shown in Fig. 4A. To begin, HfS_2_ is exfoliated onto a silicon chip with a 90-nm silica layer, and 100-nm-thick flakes are selected via AFM. The flakes are spin-coated with a negative electron-beam resist and then patterned via electron-beam lithography to define arrays of nanodisks with the target diameters [see Methods for details].

Despite the fact that HfS_2_ is a TMDC such as MoS_2_ or WS_2_, developing a dry etching recipe suitable for HfS_2_ was a key fabrication challenge, as no prior methods have been reported. We initially tested an SF_6_-based etching process commonly used for other TMDCs (*39*, *40*), but it turned out to yield a negligible etch rate. We therefore resorted to a physical etching approach using argon sputtering and optimized the etching duration to fully pattern 100-nm-thick flakes. For thicker flakes, the etching was partial, creating a different set of nanostructures (see Supplementary Text).

A dark-field optical image (at 100× magnification) of some of our fabricated HfS_2_ nanodisks is shown in Fig. 4B. In it, we observe nanodisks with nominal diameters ranging from 270 to 350 nm in steps of 10 nm. For convenience, the rest of the nanodisks, with nominal diameters ranging from 100 to 260 nm in steps of 10 nm, are not shown. An unintended consequence of our etching procedure is material redeposition, where HfS_2_ molecules removed from the flake redeposit on the sidewalls of the resist. As a result, the fabricated nanodisks exhibit slanted sidewalls rather than straight, vertical ones, with nominal diameters approximately retained at the top of each disk but widened at the base (Fig. 4, C to E). The AFM and scanning electron microscopy (SEM) images also reveal a residual thin resist layer on top of the nanodisks and that the diameter of the fabricated nanodisks is ~30 nm larger than the nominal diameter (as measured at the top surface). We have checked the difference between the nominal and real diameters of all the nanodisks and consistently found that the real diameters are ~30 nm wider.

### Resonant light scattering from HfS_2_ nanodisks

**Fig. 5.**
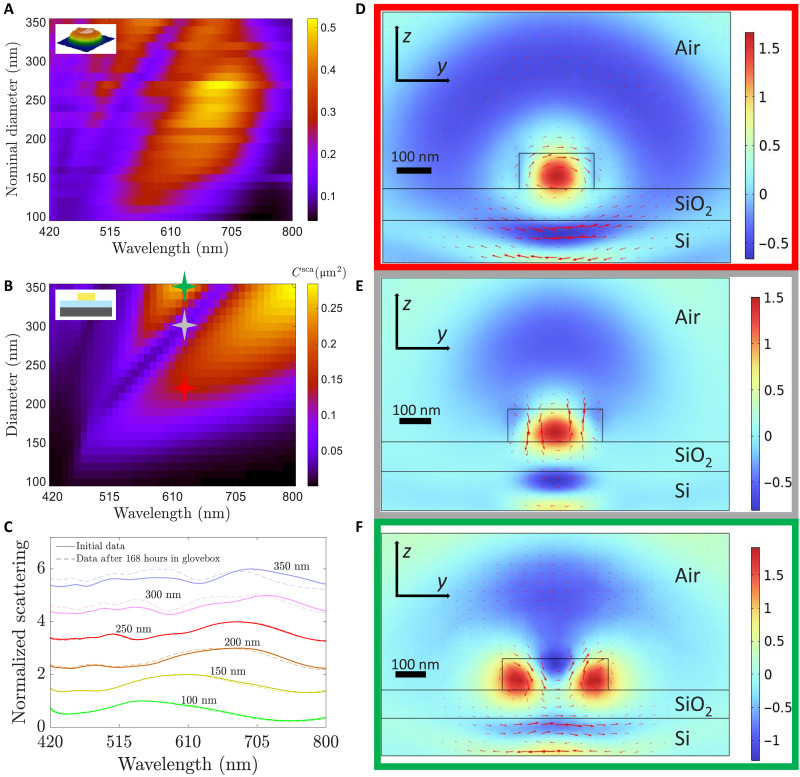
Mie resonances in HfS_2_ nanodisks. (**A**) Dark-field scattering measurements of the fabricated nanodisks (see inset). Each row of the plot corresponds to the spectral scattering measurement of a single resonator with a different nominal diameter. All of the scattering measurements are normalized by the same background signal. (**B**) Numerical scattering cross section calculated for a HfS_2_ disk with a varying diameter. The disk is placed on a 90-nm-thick layer of silica on semi-infinite silicon (see inset). The colored stars refer to three specific diameters, whose electromagnetic fields are plotted in (D) to (F) for λ = 630 nm. (**C**) Normalized dark-field scattering measurements for six nanodisks with varying diameters. The zero scattering line of each plot is moved up one unit. The solid lines are measured right after the resonators are etched, while the dashed lines are measurements after the sample has been in a glove box for 168 hours, with a total of 6 hours subjected to ambient condition. *N* = 1 for all data. (**D** to **F**) Electromagnetic scattered field at a wavelength of λ = 630 nm for diameters *d* = 210 nm, *d* = 300 nm, and *d* = 350 nm, respectively. The boxes are color-coded to match the colored stars in (B). The real part of the electric field in the $\hat{x}$ polarization is plotted as a color map, and the magnetic field is plotted as red arrows.

We conduct dark-field spectroscopy measurements on the HfS_2_ nanodisks to assess their ability to support optical resonances [see Methods], similar to nanodisks made of other high-index materials (*12*, *41*, *42*). We minimize the exposure of the nanostructures to ambient conditions by careful planning of all our experiments and storing our samples in an argon environment whenever possible. The scattering spectra of all the fabricated nanodisks sorted as a function of their nominal diameter *d* are shown in Fig. 5A. As previously mentioned, the real diameter is about 30 nm wider than the nominal diameter. All of the spectra are normalized by the same spectrum, collected from a white powder, allowing us to see how the intensity of the scattering peaks grows or gets reduced as we change the diameter of the nanodisks [see Methods]. The spectra show spectral local minima forming around *d* = 170 nm, which redshift with increasing diameters. We also observe two very well defined peak families. One of the peak families is broader than the other one, and we observe that even the smallest resonators of *d* = 100 nm can host this resonance at around a wavelength of λ = 550 nm. This resonance redshifts for larger diameters, and it also increases in intensity. The other resonance family presents sharper peaks at shorter wavelengths. The trend begins at around *d* = 200 nm for λ = 480 nm, and it slowly redshifts with increasing diameters, reaching a resonance wavelength of λ = 565 nm for a diameter of *d* = 350 nm. The redshifting of the scattering peaks for increasing resonator sizes is typically what one expects for Mie resonances.

To understand the nature of these spectral features, we conduct three-dimensional full-field electromagnetic simulations using COMSOL Multiphysics, which solves Maxwell’s equations using the finite element method. We simulate the HfS_2_ resonators as 100-nm-thick disks on top of a two-layer substrate, consisting of a 90-nm-thick layer of SiO_2_ on top of a semi-infinite silicon substrate. The system is excited with a normally incident plane wave, linearly polarized in $\hat{x}$ [see Methods]. For the refractive index of HfS_2_, we have used the experimental data shown in Fig. 2B. The computed scattering cross section (*C*^sca^) of the nanodisks is depicted in Fig. 5B, with values comparable to previous works in the field (*12*, *43*). In addition, the spectral trends of the simulated scattering cross section are very similar trends to the experimental measurements, despite (i) the different illumination conditions, normally incident plane wave in simulations versus tightly focused Bessel beam in dark-field experiment; and (ii) the idealized modeling of the HfS_2_ nanoresonators as perfectly shaped nanodisks versus nanodisks with slanted walls shown in Fig. 4 (D and E). In particular, we observe a pronounced scattering peak that forms around a wavelength of 500 nm for the smaller resonators, which redshifts as the diameter increases. The nature of this resonance can be identified by inspecting the scattered electromagnetic field, which reveals a field pattern of intense electric field in the center of the nanodisk with a magnetic field circulating around this center (Fig. 5D). Based on the field profile, we interpret this resonance as due to the excitation of an electric dipole Mie resonance. In addition to this prominent dipolar peak, we also observe that nanodisks with diameters greater than 210 nm yield a second scattering peak at shorter wavelengths. The electromagnetic field associated to this scattering peak shows a pattern with two electric field lobes at the lateral surfaces of the resonator, each of them associated with a circulating magnetic field in-phase with respect to the other (Fig. 5F). Furthermore, there is another maximum at the top surface. Given the shape of the electromagnetic field, we infer that the peak is due to a higher multipolar order, possibly a quadrupolar resonance. Notice that these two resonances are also present in the experimental results. Last, another feature that is also present in both the experiments and simulations is the spectral minima, which separate the dipolar and quadrupolar resonances. The difference between the theoretical plot and the numerical one is that the numerical plot yields a minima line that is almost linear, whereas our experimental results yield a minima line that is curved. Moreover, the experimental minima line is not as well defined as the numerical one. The field structure of this scattering minimum is depicted in Fig. 5E and resembles that of a directional forward-scatterer or photonic nanojet (*44*–*46*). Overall, we can see that our simulations capture all the prominent features of the experimental measurements.

To conclude our optical study, we compared the dark-field optical spectra of the nanodisks right after fabrication, as well as 7 days later. During these 7 days, the sample was stored in an argon-rich atmosphere in a glove box. Apart from the final spectroscopy itself, the samples were only taken out for AFM measurements. The nanodisks have been exposed to ambient conditions for ~6 hours. In Fig. 5C, we display dark-field spectra of selected nanodisks obtained right after the fabrication (solid lines), and we compare them with the spectra of the same nanodisks 7 days later (dashed lines). The plot contains spectra from structures within a wide range of nominal diameters to give a full picture of which sizes are affected the most by exposure to ambient conditions. Each spectrum is normalized to its maximum intensity value for better comparison, and the zero line is moved up one unit, so that the curves do not superimpose. This comparison shows that the nanodisks with diameters below 200 nm yield almost the same optical response after 7 days, while the larger nanodisks show moderate changes due to the exposure to ambient conditions. Notably, the spectral response of nanoresonators is much more sensitive to size or refractive index changes than that of bulk flakes. Thus, overall, we conclude that keeping the nanoresonators in an argon-rich glove box is a simple and effective way to stabilize the spectral response of the HfS_2_ nanodisks.

## DISCUSSION

This work demonstrates a rational approach to identifying and evaluating high–refractive index vdW materials by combining high-throughput DFT screening with experimental validation. The screening methodology not only highlights HfS_2_ as a promising candidate for visible-spectrum photonics but also provides a comparative overview of numerous materials with potential applications across different spectral ranges. Notably, the computational framework identifies many materials with super-Mossian behavior at lower photon energies, which could warrant further experimental exploration to expand the landscape of viable high-index dielectrics.

Focusing on HfS_2_, our study establishes it as an intriguing material for visible photonics, offering a high in-plane refractive index (>3), low optical losses, and pronounced anisotropy. These properties are leveraged to experimentally demonstrate its suitability for supporting Mie resonances in nanodisk resonators. We have shown that the chemical instability of HfS_2_ can be mitigated by storing the material in argon-rich or low-humidity environments, as well as by encapsulating it in hBN or spin-coating it with PMMA. These approaches could enhance the long-term stability of HfS_2_ and expand its practical applicability. Future work should address optimizing the fabrication process for HfS_2_-based photonic devices. While the physical etching approach used in this study successfully produced functional nanodisks, it introduced redeposition effects that could be mitigated by developing a chemical etching technique. Such improvements would enable more precise patterning and reduce fabrication-related imperfections, further unlocking the material’s potential.

Beyond HfS_2_, this study underscores the versatility of the high-throughput computational screening approach in identifying high-index optical materials. Expanding the screening to include ternary and quaternary compounds could uncover even more candidates with tailored optical and electronic properties. This methodology, which combines predictive modeling with targeted experimental validation, provides a robust framework for the rational discovery of improved optical materials. By streamlining the identification process and ensuring high-fidelity predictions, this approach can accelerate the development of new photonic materials for a wide range of technological applications.

## MATERIALS AND METHODS

### High-throughput screening

The refractive index dataset was generated using the method detailed in our previous paper in (*22*). For completeness, we reproduce the details here.

#### Electronic structure

All ground- and excited-state calculations were performed with the Atomic Simulation Environment and the GPAW electronic structure code (*47*, *48*). The atomic structure and the unit cell of the materials were relaxed until the maximum force (stress) was below 10^−4^ eV Å^−1^ (0.002 eV Å^−3^). The PBE functional for exchange and correlation effects, a Г point–centered *k*-point grid with a density of 6.0 1/Å^−1^ along each direction in the Brillouin zone, an 800-eV plane wave cutoff, and a Fermi-Dirac smearing of 50 meV, were used. VdW interactions were taken into account by the D3 correction scheme. For the most promising materials, we performed G_0_*W*_0_ calculations using the asr.gw recipe to determine their proper electronic bandgap (*49*).

#### RPA calculations for the screening

The optical permittivity, ϵ(ω) , was calculated within the RPA using the dielectric function module in GPAW. From ϵ(ω) , the refractive index and extinction coefficient were calculated as $\text{Re}\left[\sqrt{\epsilon \left(\omega \right)}\right]$ and $\text{Im}\left[\sqrt{\epsilon \left(\omega \right)}\right]$ , respectively. To ensure convergence across all materials, a *k*-point grid with a high density of 20.0 1/Å^−1^ along each direction in the Brillouin zone was used, and conduction bands up to five times the number of valence bands were included. The calculations were performed on a nonlinear frequency grid with an initial frequency spacing of 0.5 meV, a broadening of 50 meV, and a local field cutoff of 50 eV.

#### BSE+ calculations

The BSE+ method seamlessly combines a BSE description of the low-energy transitions with an RPA treatment of higher-energy transitions. As described in detail in (*24*), this is essential to obtain a quantitatively accurate description of refractive index in systems where excitonic effects are important. Because the PBE exchange correlation functional is known to underestimate electronic bandgaps, for the BSE+ calculations of HfS_2_, we apply a scissors operator to adjust the bandgaps, aligning the lowest peak observed in the refractive index calculated with BSE with the lowest peak in the refractive index observed in the experimental data. The RPA calculation was performed using 130 bands, with a local field cutoff of 80 eV. This was based on a ground-state calculation performed using the PBE functional, a plane wave cutoff of 800 eV and a Г point–centered Monkhorst-Pack 36-by-36-by-22 *k*-point grid. The BSE calculation was performed using four valence bands and three conduction bands, a local field cutoff of 80 eV, and a broadening of 0.1 eV. The electronic screening was calculated with 60 electronic bands. For the calculation of the polarizabilities in the in-plane and out-of-plane direction, Г point–centered Monkhorst-Pack grids of 12 by 12 by 6 and 12 by 12 by 8 were used, respectively.

### Imaging ellipsometry

The refractive index and extinction coefficient measurements have been done with the imaging ellipsometer Accurion EP4. We performed measurements over a broad spectral range from 360 to 1700 nm in 1-nm steps for two incident angles: 45° and 50°. For the optical modeling of ellipsometry, we used the Tauc-Lorentz oscillator approach for both in-plane and out-of-plane optical constants (*11*).

### Nanofabrication

Thin flakes were mechanically exfoliated from a HfS_2_ crystal from HQ Graphene onto a 90-nm SiO_2_ layer on top of a Si substrate. Our target was getting 100-nm-thick flakes, so we performed a number of exfoliations such that we could statistically easily obtain such thicknesses. Still, mechanical exfoliation does not allow for a precise control of thicknesses, so we used an AFM to find the flakes whose thickness is (100 ± 10) nm. For lithography, AR-N 7520 new from Allresist was used as a negative electron-beam resist. The resist was spin-coated at 2000 rpm for 60 s and postbaked at 85°C for 60 s. Then, using electron-beam lithography, we defined the mask for the nanodisks in a 30-kV Raith eLINE Plus system using a dose of 50 μC cm^−2^ and an aperture of 30 μm. Next, the resist was developed in an AR 300-47 solution from Allresist for 70 s. Last, the pillars were etched by Ar-sputtering in an ion beam etch & ion beam deposition system (IBE/IBDS) Ionfab 300 setup with a 20-mA acceleration current at a 30° incidence angle for 4 min with a flow rate of 10 SCCM (standard cubic centimeters per minute). The length of the etching was chosen so that the 100-nm-thick flakes were completely etched down, which required preliminary experiments to estimate the etch rate of Ar in each material. By fixating the samples in the etch chamber with Kapton tape, which is unaffected by the physical etch, its location could serve as a reference when measuring the post-etch height profile on the Dektak 150 profiler. The etch rate of Ar sputtering in HfS_2_ found with this method was 28.0 ± 2.2 nm/min.

### Encapsulation and spin-coating methods

#### Spin-coating

A 4 wt % solution of PMMA (996 K) in anisole was spin-coated onto the substrate at 4000 rpm for 40 s, followed by baking at 160°C for 5 min to form a uniform resist layer.

#### hBN encapsulation

The HfS_2_ flake was encapsulated using a standard hot pick-up technique, where a 7 wt % polycarbonate (PC) solution in chloroform was used as the adhesive layer on a polydimethylsiloxane base. A thin hBN flake was first picked up by heating the PC film to 130°C to promote adhesion. To transfer the hBN onto the HfS_2_ flake, the PC film was subsequently heated to 190°C, allowing it to melt and release the hBN, thereby achieving precise encapsulation of the HfS_2_. After stacking, the residual PC on the device was removed by immersing the sample in chloroform for 3 min.

### Atomic force microscopy

The thickness of the flakes and height profiles of the fabricated nanostructures were measured by a Dimension Icon-PT AFM from Bruker Analytical X-Ray Solutions in tapping mode. A scan rate of 0.5 Hz was used with at least 128 data points in each line scanned, ensuring that abrupt height changes were well resolved.

### Microscopy and optical spectroscopy

The bright- and dark-field microscopy was performed using a Nikon Eclipse LV100ND microscope and an OSL2 fiber-coupled unpolarized halogen light source for top illumination. An Andor Kymera 328i spectrograph with a slit width of 200 μm and a central wavelength of 650 nm was used to perform the reflectance and scattering spectra from the flat flakes and the nanodisks, respectively. Reflectance spectra in Fig. 3E were acquired with a 20× objective [Nikon; numerical aperture (NA) = 0.45]. The measured spectra were normalized by the reflection spectra of a protected silver mirror (Thorlabs, PF10-03-P01), which was exposed to the same illumination. For comparison purposes, we normalized all the reflectance plots to their maximum values. In contrast, the reflection measurements presented in Fig. 3F were obtained as a ratio between the reflection off the flake divided over the reflection off the substrate. The same 20× microscope objective was used. For dark-field measurements, we used a 100× objective (Nikon; NA = 0.9) to ensure maximum collection of scattered light. The disks were strategically fabricated with sufficient spacing to eliminate noise contributions from adjacent structures and were exposed long enough to have a high signal-to-noise ratio. The measured spectra were normalized by the signal collected from a Lambertian scatterer exposed to the same conditions as the sample. For each measurement, the dark counts (pixel values when no optical signal is present) of the detector were also recorded and subtracted prior to normalization. The Lambertian scatterers are Labsphere Spectralon Diffuse Reflectance Standards SRS-99-010, with a reflectance above 0.989 over the whole visible range.

### Electromagnetic simulations

We simulate the scattering response of the HfS_2_ nanodisks using COMSOL Multiphysics. A cross section of the geometry of our system can be observed in Fig. 5 (D to F). That is, we place the 100-nm-thick nanodisks on top of a 90-nm silica layer, which lies on top of a semi-infinite silicon layer. The nanodisks are surrounded by a semi-infinite layer of air. To properly model the semi-infinite air and silicon layers, we place perfectly matched layers (PMLs) on top of the air and at the bottom of the silicon layer. Moreover, we choose a material thickness of λ/2*n*, with *n* being the refractive index of the material in consideration. To be on the safe side, we implement a thickness of 400 nm for the air and a thickness of 120 nm for the silicon layer. Notice that the 400 nm of air is applied from the top of the nanodisk, which gives an air thickness of 500 nm from the silica top surface. Similar considerations are done in the transverse direction. That is, we also place PML layers on the sides of our simulations, and we make the width of the simulation to be 800 nm (one full wavelength) plus the diameter of the nanodisk, i.e., we leave λ/2 from both sides of the nanodisk walls. Even though silica and silicon have greater refractive indices, we keep the same thickness as the air. All the PMLs that we place have a thickness of λ_max_/3. We run simulations for a range of diameters varying from 100 to 350 nm in 10-nm steps. The wavelength has also been varied from 420 to 800 nm in 10-nm steps. We have computed the scattered cross section *C*^sca^ on the particle by computing the flow of the Poynting vector across the surface of the particle, which is in contact with the air domain

$$C^{\text{sca}}=\frac{\int_{S}P^{\text{sca}}\cdot dS}{I_{0}}$$ (2)

with $P^{\text{sca}}$ being the Poynting vector associated to the scattered field, and $I_{0}$ being the intensity of the incoming plane wave.
