# Supplementary material for: Computational discovery and experimental validation of high–refractive index HfS2 nanoresonators
Source: Sci Adv. 2025 Nov 5;11(45):eadw9339. doi: 10.1126/sciadv.adw9339 (PMC12588280; doi:10.1126/sciadv.adw9339)
Supplement: Supplementary file 1 — Supplementary Text Figs. S1 to S7 [file sciadv.adw9339_sm.pdf]

Science Advances
AAAS

Supplementary Materials for

Computational discovery and experimental validation of high–refractive index HfS_2 nanoresonators

Xavier Zambrana-Puyalto *et al.*

Corresponding author: Xavier Zambrana-Puyalto, xavislow@protonmail.ch;
Søren Raza, sraz@dtu.dk

Sci. Adv. **11**, eadw9339 (2025)
DOI: 10.1126/sciadv.adw9339

This PDF file includes:

Supplementary Text
Figs. S1 to S7

Supplementary Text

Anisotropy data from computational discovery

In Fig. 2A of the main body of the manuscript, we have presented a map of 72 newly discovered biaxial vdW materials, i.e., their in-plane anisotropy is below 1%. In Fig. S1, we display their static out-of-plane anisotropy, computed as $n_{\mathrm{in}} - n_{\mathrm{out}}$, as a function of their direct band gap energy. The colormap is the same as in Fig. 2A, i.e., we have used blue and gray to represent materials which are above or below the Moss line, respectively. HfS_2 has been plotted in red. In the inset of Fig. S1, we give the chemical formulas of all the super-Mossian materials. Furthermore, we have given the chemical formulas of three materials which are non super-Mossian, but whose anisotropy yields a value lower than -1, such as HgI, $IrCl_3$ and $RhCl_3$.

In Fig. S2, we plot 59 newly discovered anisotropic materials whose in-plane anisotropy is above 1%. In Fig. S2(a), we plot the static refractive index tensor in the $X - Y$ plane, with the n_{zz} values being encoded as a colormap. We give the chemical formulas of some of the vdW materials which have strong anisotropy. The $n_{\mathrm{xx}} = n_{\mathrm{yy}}$ line is plotted as a reference. The same 59 materials are plotted in Fig. S2(b), but in this case, we plot their static refractive index tensor values in 3D, and we encode their direct band gap as a colormap. We observe that the most anisotropic materials tend to have smaller band gaps. The line $n_{\mathrm{xx}} = n_{\mathrm{yy}} = n_{\mathrm{zz}}$ is also plotted as a reference.

Raman measurements

In Fig. S3, we present our results of a Raman spectroscopy study carried out with the flakes that have been used to determine the refractive index.

Reflectivity study of chemical instability

Here we present an optical study carried out with two different samples of HfS_2. We have exfoliated HfS_2 onto a substrate of SiO_2-Si, with the thickness of silica being 90 nm. The sample showed in Fig. S4(a) has been maintained in a desiccator with a simple humidity reduction down to 10%. In contrast, the central green flake shown in Fig. S4(d) has been left inside a sample box at laboratory conditions. We have measured their spectral response for a month (specifically, for 745 hrs). In Fig. S4(b-c), we plot the reflection of the HfS_2 flake shown in Fig. S4(a) divided by the reflection of

the substrate. In Fig. S4(e-f), we show the same reflection ratio for the flake that has been exposed to lab conditions. Looking at the two minima shown in Fig. S4(c) and (f), we observe that the flake shown in Fig. S4(a), which was kept in the desiccator, has the same spectral behavior throughout the month. In contrast, the flake shown in Fig. S4(d) experiences a 16 nm spectral blueshift. These results show that the chemical instability of HfS_2 can be circumvented with easily accessible storage equipment such as a desiccator. Furthermore, we also observe that the spectral shift that the flake shown in Fig. S4(d) experiences is smaller than that of the flake shown in the main text. In fact, for this case, we do not appreciate any color or structural change of the flake under the microscope. That is, the flake shown in Fig. S4(d) remains much more stable than the one present in the main text. We have not properly studied why the flake in the main text and the flake shown in Fig. S4(d) behave differently. We suspect that it is related with the fact that the relative humidity of our lab can approximately vary from 65% to 45% depending on the season, yet we leave the precise characterization of this for future work. In any case, in summary, we have found that i) HfS_2 is not necessarily as chemically unstable as reported in the main text, and ii) the chemical instability can be circumvented using very accessible storage methods such a desiccator.

Next, we present the results of another set of experiments, where we have made three samples, and kept them in a laboratory with a relative humidity which oscillates between 30% and 50%. The first sample is made of HfS_2 flakes, the second one is made of HfS_2 flakes encapsulated by a hBN layer, and the third sample consists of HfS_2 flakes with a layer of PMMA spin-coated polymer on top. Both the hBN encapsulation, and the PMMA spin-coating were carried out right after the HfS_2 flakes were exfoliated onto a silica on silicon substrate. We have measured the reflection of the three samples for a period of 647 hrs, and have divided it over the reflection of the substrate. Then, we have normalized all the data with respect to the maximum of the curve, thus yielding a normalized reflection. We present our results in Fig. S5. In Fig. S5A, we plot the normalized reflection of a flake exposed to air at two different timestamps: 1 hr (in red), and 647 hrs (in grey). We observe that, similar to Fig. S4, the reflection blueshifts for the unprotected HfS_2flake (Fig. S5(a)) . In contrast, in Fig. S5, we show the normalized reflection data of a flake encapsulated by hBN at the same timestamps. The thickness of the hBN flake stacked on top of the HfS_2 flake has not been measured. We observe that the spectrum does not shift, and the changes are negligible. Then, in Fig. S5(c), we show the normalized reflection of a HfS_2 flake which has been spin-coated with

PMMA. The measurements have been carried out at 22 hrs (green) and 647 hrs (grey) after the fabrication of the sample, respectively. Similarly to Fig. S5(b), there are no noticeable changes. Again, the thickness of the PMMA layer has not been measured. Notice that we have obtained our HfS_2 flakes via mechanical exfoliation, thus in each sample configuration there are many flakes. However, we have only tracked the reflection on one flake per configuration. Besides, the three flakes whose reflection has been tracked have different thicknesses. Their thickness has not been measured, even if given their color, we believe that the thickness is of the order of 100 nm.

Studies with non-totally etched flakes

Here, we show a parallel study that we have carried out with nano-resonators fabricated on flakes whose initial thickness was above 100 nm. As all our flakes have different thicknesses but they have the same amount of resist, the argon etching procedure yields two different types of nanostructures. In some cases, the etch caused by the Ar-sputtering was deep enough to etch the whole flake (see main text), whereas in some other cases many nanometers of material were left underneath the resonators. This is depicted in Fig. S6. The consequence of fabricating the nano-resonators on top of a flake whose thickness is above 100 nm is that we end up getting the nano-resonators on a multi-layer system composed of HfS_2-SiO_2-Si, with the thickness of HfS_2 being approximately the initial thickness of the flake minus 100 nm. In Fig. S7, we present the optical measurements carried out with these nano-resonators, which are 100 nm thick and placed on top of a 142 ± 7 nm layer of HfS_2 on the same SiO_2/Si substrate used in the main text. We can observe that this set of samples consistently yields three peaks for all nominal diameters. In particular, we observe that the peaks are almost diameter-independent, a clear departure sign of the Mie-like resonance behavior observed for the resonators in the main text. Notice that we consistently obtain resonances in the blue spectrum of the optical range, thus validating the use of HfS_2 as a potential use for photonic technologies in the visible spectrum.

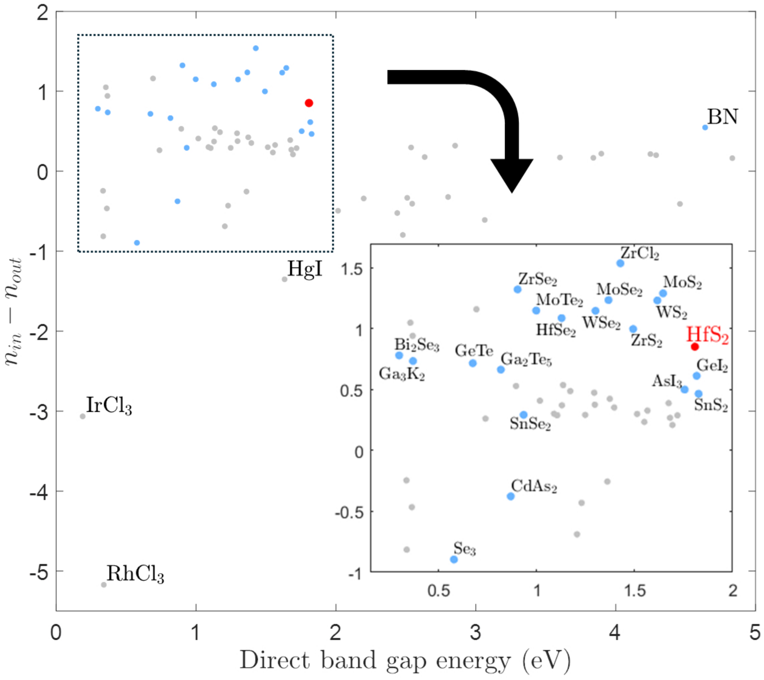

Figure S1: **Anisotropy of biaxial high-refractive-index vdW materials.** Static out-of-plane refractive index anisotropy as a function of direct band gap energy for 72 semiconductor materials with in-plane isotropy. Chemical formulas are provided for all of the super-Mossian materials, plus three others.

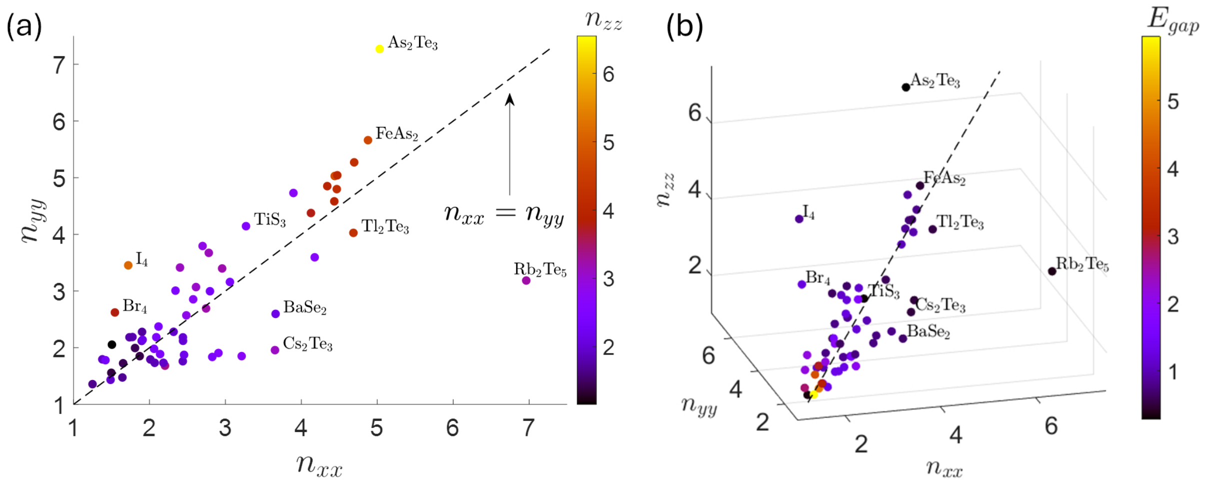

Figure S2: **Anisotropic dielectric materials.** (a) Static refractive index tensor projected in the $X - Y$ plane. The n_{zz} values are encoded as colors. (b) Static refractive index tensor projected in 3D as a function of the direct band gap, encoded as colors.

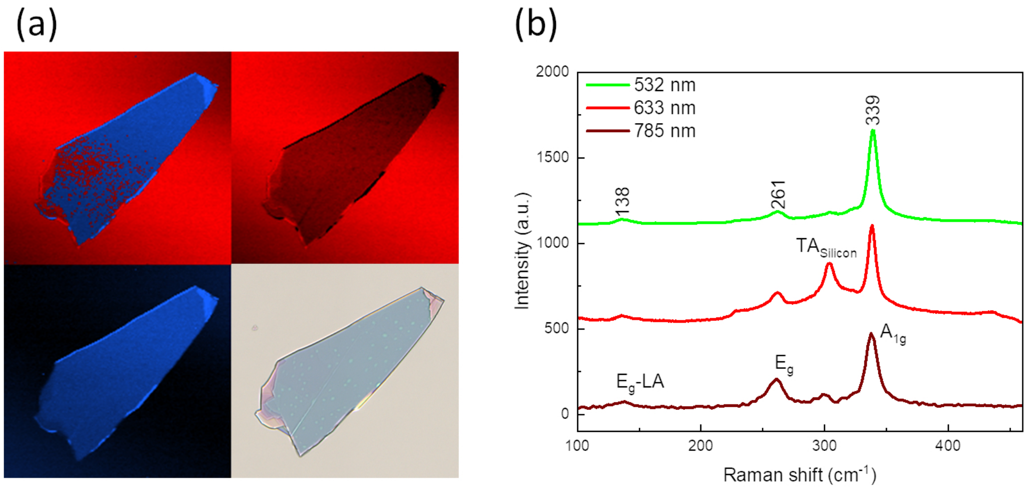

Figure S3: **Raman measurements** (a) Raman maps and optical microscopy image of a HfS_2 flake. (b) Raman spectra of HfS_2 flake.

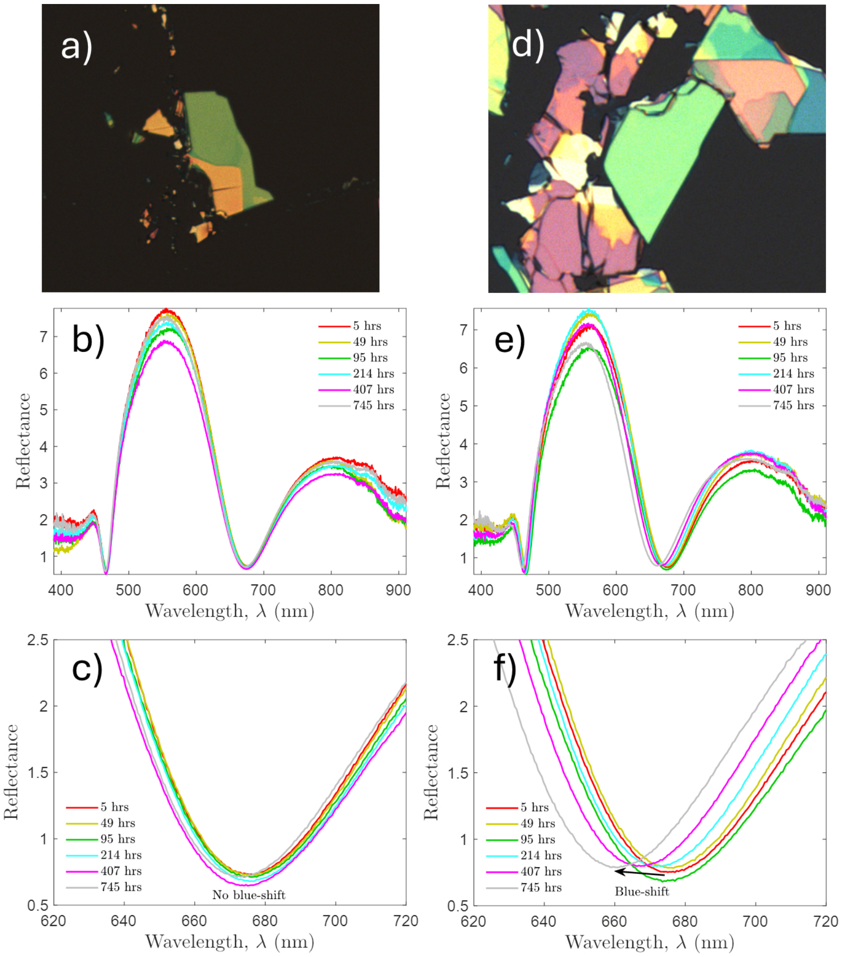

Figure S4: **Optical effects of chemical instability.** (a) In green, flake under study. The flake is left in a desiccator with a reduced humidity of 10% for 1 month. (b) Spectral behaviour of the flake shown in (a). Each line corresponds to a different measurement over time. The legend specifies the time tag (in hours) after the exfoliation procedures. (c) Expanded view of the minima of the at 680 nm. (d) In green, flake under study. The flake is left in a sample box in our optical lab for 1 month. (e) and (f) display the same measurements as (b) and (c), with the data being recorded with sample (d).

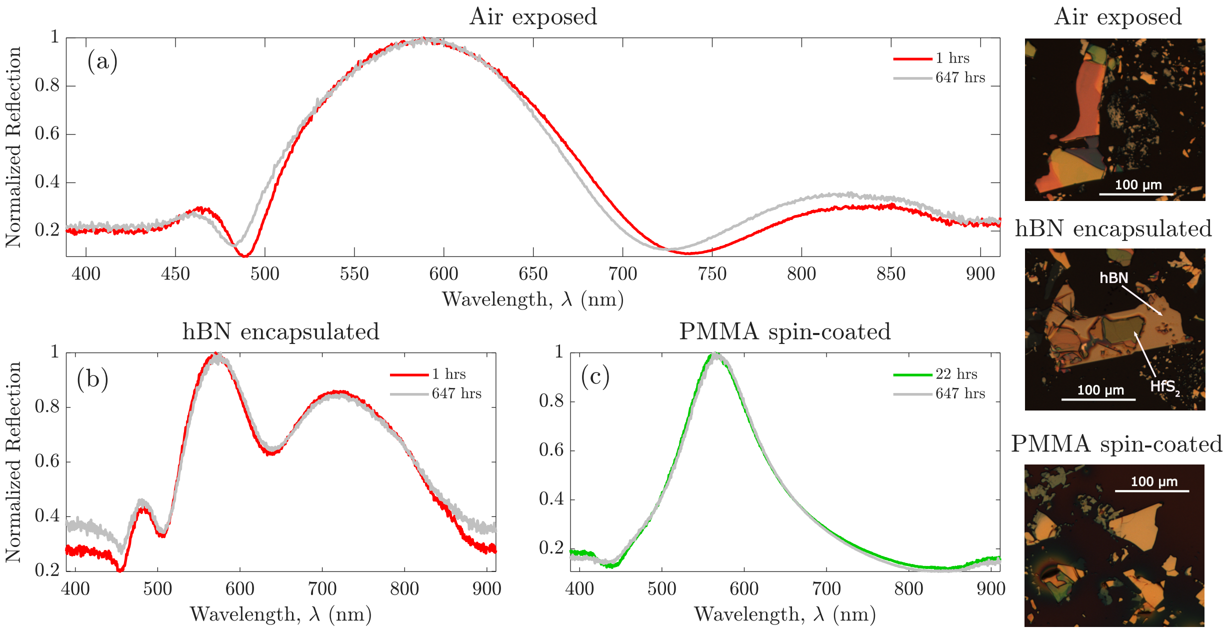

Figure S5: **Optical effects of encapsulation of HfS_2 flakes.** (a) Normalized reflection of a HfS_2 flake exposed to air 1 hr (red) and 647 hrs (grey) after exfoliation. (b) Normalized reflection of a HfS_2 flake encapsulated by hBN and exposed to air 1 hr (red) and 647 hrs (grey) after exfoliation. (c) Normalized reflection of a HfS_2 flake that has been spin-coated with PMMA 22 hrs (green) and 647 hrs (grey) after exfoliation. For all samples, the substrate onto which the flakes are exfoliated consists of 90 nm of SiO_2 on top of a Si wafer. The optical images of all the respective flakes are shown in a column on the right.

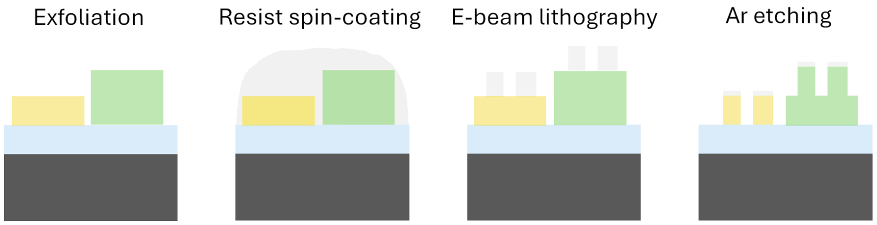

Figure S6: **Fabrication process of two different kinds of HfS_2 resonators**. (a) HfS_2 flakes are exfoliated on top of 90 nm of SiO_2 lying on top of a Si wafer. The yellow and green flakes represent a 100 and 200 nm thick flakes. (b) An ARN-7520new negative electron-beam resist is spin-coated on top of the sample. (c) The resist is exposed using e-beam lithography. The areas exposed have different areas according to the different diameters that are fabricated ranging from 100 to 350 nm in steps of 10 nm. (d) The flakes and the resist are etched using Ar-sputtering. The length of the etching is such that yellow flakes (100 nm thick) are completely etched down. The green flakes are not completely etched, thus yielding resonators on top a 3-layered system: HfS_2-SiO_2-Si.

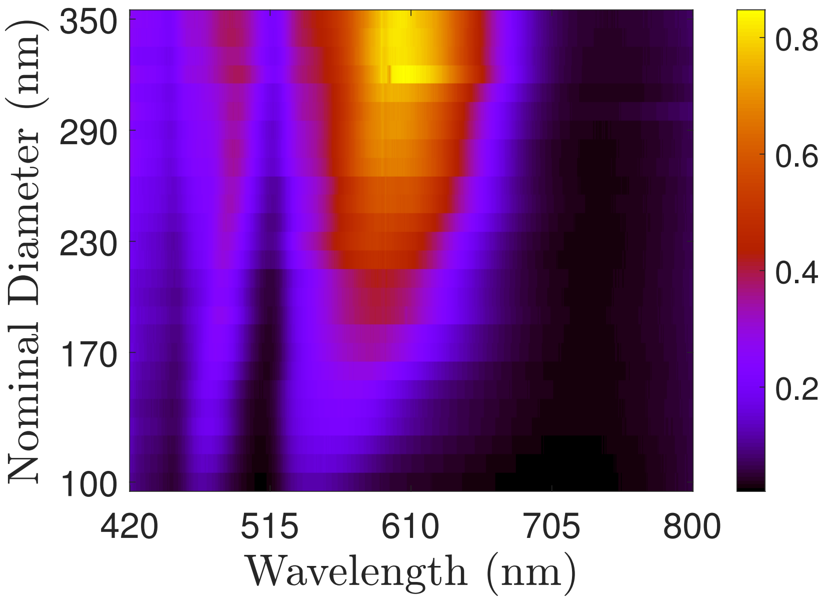

Figure S7: **Mie resonances in HfS_2 nanostructures.** Dark-field scattering measurements of the green nano-resonators in Figure S6. Each row of the plot corresponds to the spectral scattering measurement of a single resonator with a different nominal diameter. All the scattering measurements are normalized by the same background signal.
